# Tripartite motif containing 62 is a novel prognostic marker and suppresses tumor metastasis via c-Jun/Slug signaling-mediated epithelial-mesenchymal transition in cervical cancer

**DOI:** 10.1186/s13046-016-0445-5

**Published:** 2016-10-28

**Authors:** Tian-Yu Liu, Jian Chen, Chun-Liang Shang, Hong-Wei Shen, Jia-Ming Huang, Yan-Chun Liang, Wei Wang, Yun-He Zhao, Duo Liu, Man Shu, Lu-Yan Guo, Zheng Hu, Shu-Zhong Yao

**Affiliations:** 1 Department of Obstetrics and Gynecology, the First Affiliated Hospital, Sun Yat-sen University, Zhongshan Second Road 58, Guangzhou, 510800 People’s Republic of China; 2 Department of Surgery, the First Affiliated Hospital, Sun Yat-sen University, Zhongshan Second Road 58, Guangzhou, 510800 People’s Republic of China; 3 Department of Pathology, the First Affiliated Hospital, Sun Yat-sen University, Zhongshan Second Road 58, Guangzhou, 510800 People’s Republic of China

**Keywords:** Prognosis, Metastasis, EMT, MAPK, Slug

## Abstract

**Background:**

TRIM62 (tripartite motif containing 62) has been found to act as a tumor suppressor of several cancers. However, its precise biological role and related mechanism remain unknown in cervical cancer (CC).

**Methods:**

Quantitative Real-time PCR and western blot were adopted to detect the mRNA and protein expression level of TRIM62 in both human CC cell lines and tissues. Immunohistochemistry was used to measure the TRIM62 expression in 30 normal cervical and 189 CC tissues. Univariate and multivariate Cox regression analyses and Kaplan–Meier survival analyses performed to investigate the association between TRIM62 expression and CC patients’ prognosis. The effect of TRIM62 on CC growth and metastasis was studied in vitro and in vivo. Multi-pathway reporter array were utilized to identify the potential signaling manipulated by TRIM62.

**Results:**

TRIM62 was frequently down-regulated in both human CC cells and tissues. Low expression of TRIM62 in CC tissues was associated with aggressive clinicopathological features of CC patients. In addition, TRIM62 was also an independent poor prognostic factor for overall and disease-free survival of CC patients after surgery. Moreover, enforced expression of TRIM62 in CC cells significantly inhibited their abilities of proliferation, migration and invasion in vitro. Besides, subcutaneous xenograft tumor model and xenograft mouse metastatic model respectively displayed that TRIM62 impeded the growth and metastasis of CC in vivo. Furthermore, mechanism study exhibited that TRIM62 could suppress epithelial-mesenchymal transition (EMT) by inhibiting c-Jun/Slug signaling. The inhibitory role of TRIM62 in tumor proliferation might be through regulating cell cycle related proteins CyclinD1 and P27 by targeting c-Jun.

**Conclusion:**

TRIM62 is a potential prognostic biomarker in CC and suppresses metastasis of CC via inhibiting c-Jun/Slug signaling-mediated EMT.

**Electronic supplementary material:**

The online version of this article (doi:10.1186/s13046-016-0445-5) contains supplementary material, which is available to authorized users.

## Background

Cervical cancer (CC) is one of the most common malignant tumors in women. With the increasing uptake of Pap smear screening and HPV vaccines in recent years, the incidence of CC has been decreased in many countries [1]. However, there are still approximately 527,600 new cases and 265,700 deaths worldwide in 2012. Especially in developing countries, CC ranks as the second most commonly diagnosed cancer and the third leading cause of cancer death [2]. In China, there were about 98,900 new cases and 30,500 deaths among 2015 [3]. It has been well known that tumor recurrence and metastasis has become the major obstacle to improve the long-term survival of CC [4, 5]. The capability for metastasis enables CC cells to colonize new terrain where nutrients and space are more abundant than the primary site [6]. Previous researchers showed that CC metastasis is an exceedingly complex biological process, such as several oncogenes and/or anti-oncogenes aberrant expression and epithelial-mesenchymal transition (EMT) [7, 8]. The common pattern of metastasis of CC is direct extension and pelvic lymph node metastasis (PLNM), which can occur at early stage CC. In advanced CC, metastases can be present out of pelvic to distant organs, such as lungs [5]. Although persistent infection of high-risk human papillomavirus (HPV) is confirmed to be associated with the development of the majority of CC [9, 10], the molecular mechanism underlying cervical carcinogenesis and tumor progression remain unclear and there has been no accurate biomarker for predicting aggressiveness and prognosis of CC so far. [11]. As a consequence, it is of great significance to determine the exact mechanism and discover new biomarkers that are potentially able to be used in the prevention and treatment of CC.

The progression of a primary epithelial tumor to an invasive and metastatic tumor is usually accompanied by the activation of EMT, which is a biologic process that enables an epithelial cell changes to a mesenchymal cell phenotype [12, 13]. A number of studies have found EMT could endow epithelial cell with migratory and invasive capacity, and was involved in metastasis of many cancers including CC [14, 15]. EMT is induced by a group of transcription factors such as Snail, Slug and Twist. These transcription factors can mediate the formation of a positive feedback loop via activating the expression of either their own or other EMT transcription factors, consequently activating the cascade of EMT [13]. The expression of EMT transcription factors can be activated by multiple cellular pathways, for instance MAPK/JNK signaling. Activator protein-1 (AP-1) is one of the major downstream transcription factors of MAPK/JNK signaling. It is a dimeric complex, which comprise three main subfamilies: Jun (c-Jun, JunB, JunD), Fos (c-Fos, FosB, Fra-1, Fra-2) and ATF [16]. As a component of AP-1, c-Jun can bind to the Slug promoter and increase Slug expression, consequently inducing the program of EMT [13, 17].

As a member of the TRIM/RBCC (tripartite motif or RING finger, B-box and coiled-coil) family, TRIM62 (tripartite motif containing 62) is a RING finger E3 ubiquitin ligase [18]. Also known as DEAR1 (ductal epithelium-associated RING chromosome 1), TRIM62 has the function of regulating cell polarity and epithelial plasticity [19]. In 2009, Chen et al discovered that TRIM62 was a dominant regulator of acinar morphogenesis in the mammary gland [20]. Later in 2013, they further elucidated the function of TRIM62, which acted as a chromosome 1p35 tumor suppressor and negative regulator of TGFβ-driven EMT [21]. It is reported that TRIM62 was highly expressed in normal epithelia, but mutations were identified in several epithelial cancers, including breast cancer, lung cancer, and ovarian cancer [20–23]. Moreover, studies of breast cancer, lung cancer and acute myeloid leukemia all demonstrated TRIM62 was a tumor suppressor and its low expression was correlated with poor clinical prognosis [20, 23, 24]. However, the role of TRIM62 in CC has never been explored.

In this study, we explored the expression and biological functions of TRIM62 in CC and sought to identify the involved mechanisms for the first time. Our results demonstrated that TRIM62 was frequently down-regulated in both CC tissues and cell lines. Further analysis indicated that it was an independent predictor of both overall survival (OS) and disease-free survival (DFS) in early-stage CC. In addition, in vitro and in vivo investigations showed that TRIM62 significantly inhibited proliferation, growth and metastasis of CC. Furthermore, mechanism study found that the inhibitory role of TRIM62 in tumor metastasis was due to suppression of c-Jun/Slug signaling-mediated EMT. Its inhibitory role of proliferation might through regulating cell cycle related proteins CyclinD1 and P27 by targeting c-Jun in CC.

## Methods

### Patients and tissue specimens

For detecting both the messenger RNA (mRNA) and protein expression level of TRIM62 in cervical tissue, 20 normal cervical tissue (NCT) samples and 40 early-stage CC tissue (28 squamous cell carcinoma and 12 adenocarcinoma) samples were selected for quantitative real-time PCR (qRT-PCR). From above-mentioned tissues, eight NCT and eight CC tissues (4 squamous cell carcinoma samples and 4 adenocarcinoma samples) were selected for western blot. All samples were treated with liquid nitrogen freezing and stored at -80 °C for later RNA or protein extraction. Moreover, six early-stage CC tissue samples and paired adjacent noncancerous cervical tissue (ANT) samples were collected for immunohistochemistry. For further study, our research enrolled a total of 426 patients diagnosed with CC who underwent radical hysterectomy and lymphadenectomy in the Department of Gynecology and Obstetrics, the First Affiliated Hospital of Sun Yat-sen University from January 2003 to December 2010. The training cohort contained randomly selected 108 cases in 217 patients from January 2007 to December 2010. The validation cohort contained randomly selected 81 cases in 162 patients from January 2003 to December 2006 (Additional file 1: Figure S1). Thirty NCT samples were adopted as normal control. TRIM62 expression in all the above-mentioned tissues was detected by IHC. Histopathology was evaluated by two pathologists in the Department of Pathology at the First Affiliated Hospital of Sun Yat-sen University. The comparison of the clinicopathological features between two cohorts showed no statistically significant differences (Additional file 2: Table S1). All research protocols strictly complied with REMARK guidelines for reporting prognostic biomarkers in cancer [25]. All the enrolled CC patients were in the Ia2–IIa2 stage (early-stage). All patient materials were obtained with informed consents. This study was approved by the Ethics Committee of the First Affiliated Hospital of Sun Yat-sen University.

### Cell lines and cell culture

In this study, eight human cervical cancer cell lines were used, including SiHa, HeLa, CaSki, ME180, HCC94, HeLa229, MS751 and C33A. Among the cells above: SiHa, HeLa, CaSki, ME180 and C33A cells were kindly gifted by the State Key Laboratory of Oncology in South China. HCC94, HeLa229 and MS751 were obtained from the Type Culture Collection of the Chinese Academy of Sciences, Shanghai, China. The cell lines SiHa, HeLa and ME180 were cultured in DMEM medium (Gibco BRL, Rockville, MD). The cell lines CaSki, HCC94 and HeLa229 were cultured in RPMI-1640 medium (Gibco BRL). The cell lines C33A and MS751 were cultured in Eagle’s minimum essential medium (Gibco BRL). Media were supplemented with 10 % fetal bovine serum (HyClone Laboratories, Logan, UT) and 1 % antibiotics (100 U/ml penicillin and 100 μg/ml streptomycin) in a 5 % CO_2_-humidified atmosphere at 37 °C.

### RNA isolation and quantitative real-time PCR

Total RNA was isolated using RNAiso plus reagent (TAKARA, Dalian, China) according to the manufacturer’s protocol and total RNA of each sample was measured quantitatively by NanoDrop ND-1000. RNA was reverse-transcribed into cDNA by using PrimeScript RT Master Mix (TAKARA, Dalian, China). The quantitative real-time PCR (qRT-PCR) analyses were performed on a 7500 fast Real-Time PCR system (Applied Biosystems, USA) utilizing SYBR Premix Ex Taq (TAKARA, Dalian, China). In the PCR cycling (40 cycles), pre-denaturation was accomplished in 30s at 95 °C,while the parameters for denaturation and annealing was set at 95 °C, 5 s and 60 °C, 34 s separately. The qRT-PCR primer sequences of *TRIM62* were referenced as follows [20]: forward, 5′-TTGATCCAAGGATGTGACATG-3′ and reverse, 5′-GTGACCACTGTGGACTGGG-3′. The qRT-PCR was repeated at least three times. Relative fold changes of expression in tumor tissues against normal cervical tissue and among different cell lines were calculated using the comparative Ct (2^-△△Ct^) method. Expression data were normalized to the geometric mean with reference to the housekeeping gene β-actin.

### Western blot

Total proteins of cell lines, fresh tissue and xenografts were extracted with cold RIPA lysis buffer supplemented with protease inhibitor. Total proteins were separated by sodium dodecyl sulfate-polyacrylamide gel electrophoresis (SDS-PAGE) and then transferred onto the PVDF membrane (Roche Life Sciences, Switzerland) as previously described. The membrane were blocked with 5 % skimmed milk and incubated with the appropriate antibody. The antigen-antibody complex on the membrane was detected with Pierce ECL Western Blotting Substrate (Thermo Scientific, Waltham, MA). The antibodies are listed in the Additional file 2: Table S6.

### Immunohistochemistry

IHC staining was performed in the paraffin-embedded tissue samples cut in 4-cm sections. First deparaffinized in xylene and rehydrated using a series of graded alcohols, then slides were blocked with 10 % goat serum before incubating with a primary antibody overnight, followed by HRP conjugated secondary antibody incubation for 30 min at room temperature. Antibody binding was detected by DAB and reaction was stopped by immersion of tissue sections in distilled water once brown color appeared. Tissue sections were counterstained by hematoxylin, dehydrated in graded ethanols and mounted. The antibodies were listed in the Additional file 2: Table S6. The positive level of immunohistochemical staining was scored as described [26]. For statistical analysis, the IHC scores (ranging from 0 to 6) were evaluated and the staining score of 4 was defined as the cutoff. Thus, patients with different positive level of TRIM62 expression were divided into low- and high-staining groups. The representative images of different TRIM62 expression level were shown in Fig. 2a.

### Lentivirus vectors construction and transfection

To up-regulate TRIM62 expression, the PCR-amplified human TRIM62 coding sequence was inserted into the NotI/BamHI site of lentivirus expression vector EF-1aF/GFP&Puro, and then transfected into SiHa and HeLa cells. The rescue of c-Jun expression via lentivirus transfection was conducted likewise. Meanwhile the empty lentivirus vectors transfected into SiHa and HeLa cells were used as the negative controls. All of these lentivirus systems were purchased from GeneChem (Shanghai, China). Stable cell lines were selected for 10 days with 0.5 mg/ml puromycin 48 h after infection.

### CCK-8 assays

To test cell viability, CCK-8 assays were performed as the following method. The stable cell lines SiHa-NC, SiHa-TRIM62, HeLa-NC, and HeLa-TRIM62 were counted and 2 × 10^3^ stably infected cells were seeded into each well of 96-well plates. Cell viability was determined by the Cell Counting Kit-8 (CCK-8, DOJINDO, Japan) and the microplate reader (Tecan Sunrise, Tecan Group Ltd.) at a wavelength of 450 nm.

### Colony formation assays

For colony formation assays, cells were seeded at a density of 500 cells per 35 mm culture dish (Corning Costar Corp, Corning, NY). Then the cells were continuously cultured for 2 weeks. Subsequently, we removed the medium and stained the cells with crystal violet (KeyGEN biotech, Nanjing, China). Visible colonies (>50 cells/colony) in the dishes were manually counted and compared. All experiments were performed independently in triplicate.

### Flow cytometry assays

For flow cytometry assays, 1 × 10^6^ cells were harvested and washed twice with cold PBS, followed by fixation with 70 % ethanol 24 h at 4 °C. Cell cycle analysis was performed according to the manufacturer’s protocol of a cell cycle detection kit (Beyotime Institute of Biotechnology, Beijin, China). The samples were analyzed using a Gallios flow cytometer (Beckman Coulter), and the cell cycle distribution was analyzed by FlowJo software.

### Transwell assays

Cells in culture dish were preincubated with Mitomycin-C (10 μg/ml) for 1 h at 37 °C to suppress proliferation. For the transwell migration assay, 2 × 10^4^ cells in 100 μl serum-free medium were seeded into the upper chamber of 8-μm transwell inserts (BD Biosciences, Franklin Lakes, NJ). In the lower chamber 500 μl medium containing 10 % bovine serum albumin was added. After 20 h of incubation at 37 °C, cells in the upper chamber were removed carefully. While cells adhering to the underside of the membrane were fixed in methanol for 15 min and then stained with 0.1 % crystal violet (KeyGEN biotech) for 30 min. For the transwell invasion assays, 2 × 10^5^ cells in 100 μl serum-free medium were put into the upper chamber, which pre-coated with 50 μl Matrigel (BD Biosciences, Bedford, MD) diluted 1:4 with serum-free medium. While medium containing 10 % bovine serum albumin were in the lower chamber. After 24 h of incubation at 37 °C, Matrigel and cells in the upper chamber were removed carefully, while cells adhering to the underside of the membrane were fixed in methanol for 15 min and then stained with 0.1 % crystal violet (KeyGEN biotech) for 30 min.

The number of cells was counted in 5 randomly selected visual fields under an inverted microscope DMI4000B (Leica, Wetzlar, Germany).

### Immunofluorescence

With the purpose of cytoskeleton analysis, F-actin staining using rhodamine-phalloidin was performed. Processing for immunofluorescence, cells which grown on glass coverslips were washed twice with preheated (37 °C) PBS for 5 min each time and fixed for 10 min in 3.7 % formaldehyde dissolved in PBS. Then cells were permeabilized with 0.1 % Triton in PBS for 5 min and blocked with 1 % bovine serum albumin (BSA) in PBS for 15 min. F-actin was stained with rhodamine-phallotoxin (Sigma, St. Louis, MO) in PBS containing 1 % BSA for 40 min at room temperature as the manufacturer’s protocol. Wash several times with PBS to remove unbound rhodamine-phalloidin. Finally cells were incubated with DAPI (Roche Life Sciences, Switzerland) and then washed three times with PBS for 5 min each time. The samples were imaged by an automatic fluorescence microscope BX63 (Olympus, Wetzlar, Germany).

### Subcutaneous xenograft tumour model and xenograft mouse metastatic model

In order to test the ability of forming tumors in vivo, subcutaneous xenograft tumour model, which injecting stable cells into nude mice, was performed. Mice were bred and maintained under SPF conditions in the Department of Sun Yat-sen University Animal Center, as approved by the China Care Committee Institute. The female BALB/c nude mice (4–6weeks of age, 18–20 g) were randomly divided into 4 groups (*n =* 6/group). The stable cells (5 × 10^6^) including SiHa-NC, SiHa-TRIM62, HeLa-NC, and HeLa-TRIM62 cells were injected subcutaneously into the lower back of the female BALB/c nude mice. After injection xenograft tumours were examined twice weekly. Tumors’ length and width was measured using calipers and volumes were calculated according to the formula: length × width^2^ × 0.52, as described previously [27]. All mice were sacrificed at the 30th day after injection. Tumors were removed from each mouse carefully, weighed and paraffin embedded. Serial 6.0 mm sections were cut and subjected to immunohistochemical staining.

Xenograft mouse metastatic model was performed for comparing the metastatic capability between different stable cell lines (SiHa-NC vs SiHa-TRIM62, HeLa-NC vs HeLa-TRIM62). The cells (2 × 10^6^/150 μL per mouse) were injected intravenously into the tail vein of female BALB/c-nu mice, respectively. After 6 weeks, the mice were sacrificed and the lungs were removed, fixed with 3.7 % formaldehyde for Hematoxylin and Eeosin (H&E) staining. The number of mice pulmonary metastatic foci was confirmed and recorded by specialized pathologists.

### Multi-pathway reporter array

A Cignal Finder 10-Pathway Reporter Array (SABiosciences, QIAGEN, USA) and Dual-Luciferase® Reporter Assay System (Promega, USA) were adopted for the pathway analysis. Transient transfection was performed using Lipofectamine LTX and PLUS™ Reagents (Invitrogen, CA, USA). Both plasmid DNAs for the respective signaling pathways provided in the kit as well as Lipofectamine LTX and PLUS™ Reagents were diluted using Opti-MEM (Invitrogen, USA). Relative firefly luciferase activity was calculated and normalized to the constitutively expressed Renilla luciferase. The assay was performed as protocols provided in the kits.

### Statistical analysis

Statistical analyses were performed using the SPSS 13.0 statistical software (Chicago, IL, USA) and MedCalc statistical software (Mariakerke, Belgium). The differences between groups were analyzed by Student’s t test. The χ^2^ test and Fisher’s exact test were used to analyze the relationship between TRIM62 expression and the clinicopathological characteristics. Survival data were evaluated using univariate and multivariate Cox regression analyses. Survival curves were plotted by the Kaplan–Meier method and compared using the log-rank test. In all cases, *P* < 0.05 was considered statistically significant.

## Results

### TRIM62 is frequently down-regulated in human cervical cancer tissues and cell lines

**Fig. 1:**
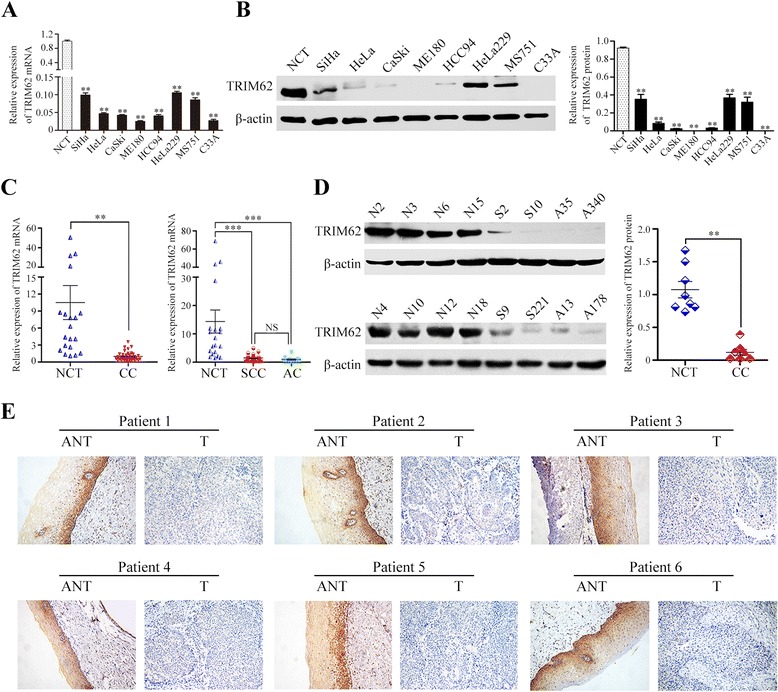
The protein and mRNA expression of TRIM62 determined by western blot, qRT-PCR and IHC assays. The expression level of TRIM62 was normalized to β-actin. The error bars represent standard deviation values calculated from three parallel experiments. NCT, normal cervical tissue; CC, cervical cancer; SCC, squamous cell carcinoma; AC, adenocarcinoma; NS, no statistical significance; **, *P* < 0.01; ***, *P* < 0.001. **a** Analysis of the mRNA expression level of TRIM62 in eight human CC cell lines by qRT-PCR. **b** Western blot analyzed the protein expression level of TRIM62 in eight human CC cell lines (*left*). Quantitative analysis of TRIM62 protein expression in eight human CC cell lines was displayed (*right*). **c** Utilizing qRT-PCR assays, TRIM62 mRNA expression were detected in 20 NCT and 40 early-stage CC tissues. There were 28 SCC and 12 AC tissues. **d** Western blot assay of TRIM62 protein expression were detected in 8 NCT and 8 early-stage CC tissues, including 4 SCC and 4 AC tissues (*left*). Quantitative analysis of TRIM62 protein expression in NCT and CC tissues was shown (*right*). **e** IHC assays were performed for investigating TRIM62 protein expression in six pairs of matched cervical cancer (T) samples and adjacent nontumor cervical tissue (ANT) samples. The TRIM62 expression located in the cytoplast was strong in NCT of ANT samples, weak or negative in T samples. Original magnifications: ×200

To explore the protein and mRNA expression of TRIM62 in CC cell lines, we performed western blot and qRT-PCR analyses on eight human CC cell lines, namely SiHa, HeLa, CaSki, ME180, HCC94, HeLa229, MS751 and C33A. TRIM62 mRNA was down-regulated by 9.4 to 39.4 fold in all CC cell lines compared to NCT (Fig. 1a). In consistence with these data, TRIM62 protein was also absent or markedly decreased in 62.5 % (5/8) of the CC cell lines assessed, including those derived from highly invasive and/or metastatic CC (HeLa, CaSki, ME180) (Fig. 1b).

To determine whether TRIM62 was also lowly expressed in CC tissues, firstly 20 NCT and 40 early-stage CC tissues were selected for qRT-PCR. Meanwhile, eight NCT and eight early-stage CC tissues (4 squamous cell carcinoma samples and 4 adenocarcinoma samples) were selected randomly from above-mentioned tissues for western blot. The results showed that TRIM62 was less expressed in early-stage CC tissues, compared with NCT, both protein and mRNA level. (Fig. 1c and Fig. 1d). The expressions of TRIM62 mRNA were markedly down-regulated in both cervical squamous cell carcinoma (SCC) and adenocarcinoma (AC), respectively in comparison with those in NCT (Fig. 1c). However, the mRNA expression of TRIM62 in SCC and in AC demonstrated no statistical difference (*P* > 0.05) (Fig. 1c). Moreover, six early-stage CC tissue samples and paired ANT samples were performed for IHC. Compared with matched ANT, the expression of TRIM62 in all six CC samples was markedly down-regulated (Fig. 1e).

**Fig. 2:**
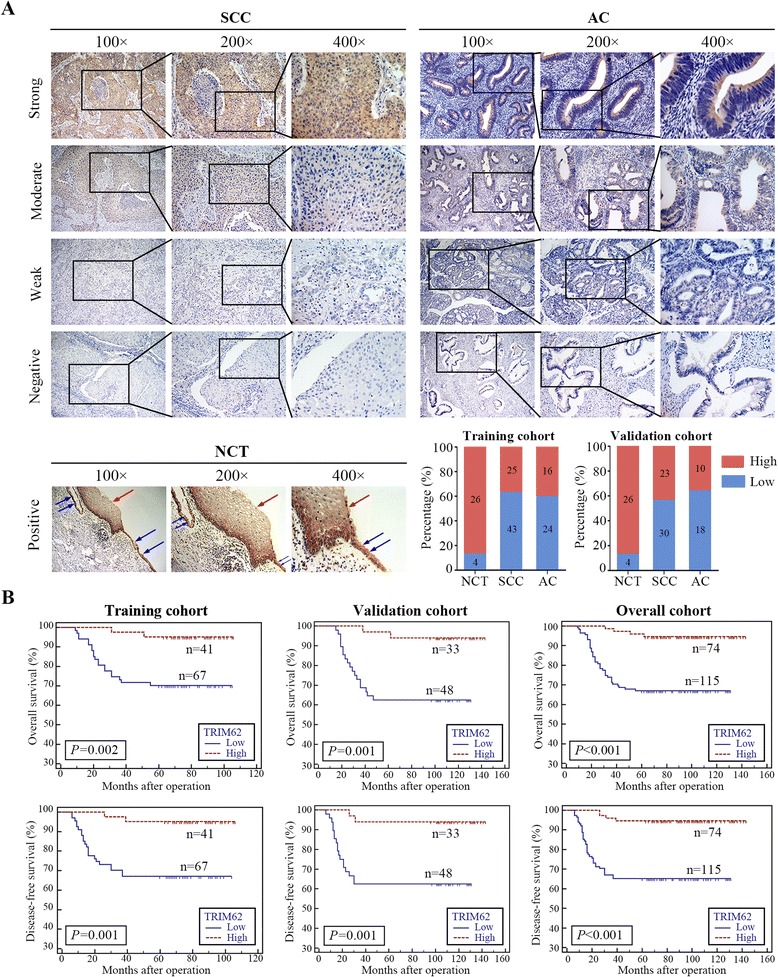
TRIM62 was frequently down-regulated in CC and was significantly correlated with overall survival and disease-free survival. NCT, normal cervical tissue; CC, cervical cancer; SCC, squamous cell carcinoma; AC, adenocarcinoma. **a** Representative IHC images of CC tissues (SCC and AC) with different staining intensity (*upper*). Representative IHC images of NCT were conducted as control (*lower*), in which both squamous and glandular epithelial cells were stained throughout the cytoplasm, and the red arrows indicated the squamous epithelial cells and the blue arrows indicated the glandular epithelial cells. Original magnification: ×100, ×200 and × 400. Bar graph shows statistics for high/low expression of NCT, SCC and AC in the training and validation cohorts. **b** Survival curve was calculated with Log-rank test. The results showed the overall survival and disease-free survival of patients with high or low TRIM62 expression in the training cohort, validation cohort and overall cohort. *P* value was shown in each panel respectively

Furthermore, to validate the results above, 189 cases of early-stage CC were involved for IHC. These cases were divided into a training cohort and a validation cohort as it was described in Materials and Methods. For the training cohort, TRIM62 expression was remarkably down-regulated compared with NCT (Fig. 2a). The results showed that 62.0 % (67/108) of the specimens had low expression of TRIM62 (Fig. 2a and Table 1). In consistence with the results of training cohort, 59.3 % (48/81) of the specimens showed low expression of TRIM62 in the validation cohort. (Fig. 2a and Table 1). Taken together, in 189 cases of the overall cohort, 115 out of 189 specimens showed low expression of TRIM62 (Additional file 2: Table S2).

**Table 1:** Correlation between TRIM62 Expression and Clinicopathologic Characteristics of Early-stage CC in Training Cohort and Validation Cohort

Clinicopathologic Variable	Training Cohort				Validation Cohort			
	Total	TRIM62 expression		*P*-value	Total	TRIM62 expression		*P*-value
		low	High			low	High	
Age(years)								
≦42	61	40	21	0.388	44	26	18	0.973
> 42	47	27	20		37	22	15	
FIGO stage								
Ia2	9	2	7	0.063	11	2	9	***0.007*** ^***a***^
Ib1	56	34	22		39	22	17	
Ib2	18	12	6		14	9	5	
IIa1	12	8	4		7	6	1	
IIa2	13	11	2		10	9	1	
Tumor size(cm)								
≦4	74	41	33	***0.036***	54	27	27	***0.016***
> 4	34	26	8		27	21	6	
Pathologic types								
Squamous cell carcinoma	68	43	25	0.738	53	30	23	0.503
Adenocarcinoma	40	24	16		28	18	10	
Differentiation grade								
Well	9	4	5	0.081	7	3	4	***0.046*** ^***a***^
Moderate	48	26	22		36	17	19	
Poor	51	37	14		38	28	10	
Stromal invasion								
< 1/2	61	30	31	***0.004***	50	25	25	***0.031***
≧1/2	47	36	11		31	23	8	
Lymphovascular space invasion								
Yes	14	12	2	0.050	11	8	3	0.511^a^
No	94	55	39		70	40	30	
Pelvic-lymph node metastasis								
Yes	18	15	3	***0.041***	14	11	3	0.106
No	90	52	38		67	37	30	
Vaginal involvement								
Yes	4	4	0	0.295^a^	3	3	0	0.267^a^
No	104	63	41		78	45	33	
Parametrial infiltration								
Yes	3	3	0	0.287^a^	2	2	0	0.511^a^
No	105	64	41		79	46	33	
Recurrence								
Yes	24	22	2	***0.001***	20	18	2	***0.001***
No	84	45	39		61	30	31	
Vital status at follow-up								
Alive	86	47	39	***0.002***	61	30	31	***0.001***
Dead	22	20	2		20	18	2	

Abbreviations: *FIGO* the International Federation of Gynecology and Obstetrics

^a^ *P*-value from Fisher’s exact test; The bold number inside the table reflected *P* < 0.05

All these data suggested that the aberrant down-regulated expression of TRIM62 was a frequent event in early-stage CC.

### Low expression of TRIM62 in early-stage cervical cancer tissue correlates with poor clinicopathologic features and prognosis

According to the expression of TRIM62 in early-stage CC tissues, all of the patients were divided into a low expression group (IHC score < 4) and a high expression group (IHC score ≥ 4). Then the relationship between the expression of TRIM62 and clinicopathologic features in the training cohort, the validation cohort and the overall cohort were analyzed respectively. Significant correlations were identified between low TRIM62 expression and several poor clinicopathologic features in the training cohort (Table 1), including tumor size (*P* = 0.036), stromal invasion (*P* = 0.004), PLNM (*P* = 0.041), recurrence (*P* = 0.001) and vital status at follow-up (*P* = 0.002). There was no obvious relationship with age, FIGO stage, pathologic types, differentiation grade, lymphovascular space invasion (LVSI), vaginal involvement and parametrial infiltration (Table 1). In the validation cohort, similar correlation results were found. TRIM62 expression was associated with tumor size (*P* = 0.016), stromal invasion (*P* = 0.031), recurrence (*P* = 0.001) and vital status at follow-up (*P* = 0.001) (Table 1). However, TRIM62 expression in the validation cohort was also associated with FIGO stage (*P* = 0.007) and differentiation grade (*P* = 0.046), but not with PLNM, which were different with that of the training cohort. Except above results, there was no obvious relationship with age, pathologic types, LVSI, vaginal involvement and parametrial infiltration (Table 1). To prove whether TRIM62 is an independent risk factor, univariate and multivariate analyses were carried out. Our data showed that, in training cohort, TRIM62 expression (*P* = 0.032) and FIGO stage (*P* = 0.011) were independent prognostic factors for overall survival (OS) (Table 2). TRIM62 expression (*P* = 0.017) and FIGO stage (*P* = 0.008) were independent prognostic factors for disease-free survival (DFS) (Table 2). Similarly, in the validation cohort, TRIM62 expression (*P* = 0.038) and FIGO stage (*P* = 0.017) were independent prognostic factors for OS (Table 3). TRIM62 expression (*P* = 0.036) and FIGO stage (*P* = 0.013) were independent prognostic factors for DFS (Table 3). Furthermore, in the training cohort, Kaplan–Meier survival curves and the log-rank test survival analysis (Fig. 2b) showed that patients in the low TRIM62 expression group had shorter OS and DFS than that in the high expression group (*P* = 0.002 and *P* = 0.001, respectively). In the validation cohort, similar results were found that low TRIM62 expression group had shorter OS and DFS than that in the high expression group (*P* = 0.001 and *P* = 0.001, respectively) (Fig. 2b).

**Table 2:** Univariate and Multivariate Analysis of Factors Associated with Overall Survival and Disease-Free Survival in Training Cohort

Clinicopathologic Variable	Total	OS			DFS		
		Univariate	Multivariate Analysis		Univariate	Multivariate Analysis	
		*P*	RR (95 % CI)	*P*	*P*	RR (95 % CI)	*P*
Age(years)							
≦42	61						
> 42	47	0.424	n.a.	n.a.	0.454	n.a.	n.a.
FIGO stage							
Ia2	9		1			1	
Ib1	53						
Ib2	21						
IIa1	12						
IIa2	13	***<0.001***	1.697(1.128–2.555)	***0.011***	***<0.001***	1.681(1.148–2.463)	***0.008***
Tumor size(cm)							
≦4	74		1				
> 4	34	***0.035***	0.650(0.203–2.078)	0.467	0.068	n.a.	n.a.
Pathologic types							
Squamous cell carcinoma	68						
Adenocarcinoma	40	0.688	n.a.	n.a.	0.921	n.a.	n.a.
Differentiation grade							
Well	9						
Moderate	48						
Poor	51	0.209	n.a.	n.a.	0.117	n.a.	n.a.
Stromal invasion							
< 1/2	61		1			1	
≧1/2	47	***0.011***	1.740(0.535–5.662)	0.357	***0.012***	1.327(0.507–3.474)	0.565
Lymphovascular space invasion							
Yes	14					1	
No	94	0.115	n.a.	n.a.	***0.046***	0.629(0.190–2.080)	0.447
Pelvic-lymph node metastasis							
Yes	18		1			1	
No	90	***0.029***	1.609 (0.646–4.011)	0.307	***0.014***	2.211(0.788–6.206)	0.132
Vaginal involvement							
Yes	4						
No	104	0.824	n.a.	n.a.	0.862	n.a.	n.a.
Parametrial infiltration							
Yes	3						
No	105	0.434	n.a.	n.a.	0.505	n.a.	n.a.
TRIM62							
low	67		1			1	
high	41	***0.008***	0.200(0.046–0.873)	***0.032***	***0.005***	0.168(0.039–0.728)	***0.017***

Abbreviations: *n.a.* Not application, *FIGO* the International Federation of Gynecology and Obstetrics; The bold number inside the table reflected *P *< 0.05

**Table 3:** Univariate and Multivariate Analysis of Factors Associated with Overall Survival and Disease-Free Survival in Validation Cohort

Clinicopathologic Variable	Total	OS			DFS		
		Univariate	Multivariate Analysis		Univariate	Multivariate Analysis	
		*P*	RR (95 % CI)	*P*	*P*	RR (95 % CI)	*P*
Age(years)							
≦42	44						
> 42	37	0.682	n.a.	n.a.	0.726	n.a.	n.a.
FIGO stage							
Ia2	11		1			1	
Ib1	39						
Ib2	14						
IIa1	7						
IIa2	10	***<0.001***	1.546(1.082–2.211)	***0.017***	***<0.001***	1.575(1.102–2.250)	***0.013***
Tumor size(cm)							
≦4	54						
> 4	27	0.191	n.a.	n.a.	0.210	n.a.	n.a.
Pathologic types							
Squamous cell carcinoma	53						
Adenocarcinoma	28	0.933	n.a.	n.a.	0.908	n.a.	n.a.
Differentiation grade							
Well	7						
Moderate	36						
Poor	38	0.105	n.a.	n.a.	0.115	n.a.	n.a.
Stromal invasion							
< 1/2	50		1			1	
≧1/2	31	***0.020***	1.900(0.763–4.734)	0.168	***0.024***	1.819(0.735–4.502)	0.196
Lymphovascular space invasion							
Yes	11						
No	70	0.122	n.a.	n.a.	0.182	n.a.	n.a.
Pelvic-lymph node metastasis							
Yes	14		1			1	
No	67	***0.016***	2.098(0.821–5.358)	0.122	***0.026***	1.775(0.697–4.518)	0.229
Vaginal involvement							
Yes	3						
No	78	0.769	n.a.	n.a.	0.662	n.a.	n.a.
Parametrial infiltration							
Yes	2						
No	79	0.218	n.a.	n.a.	0.316	n.a.	n.a.
TRIM62							
low	48		1			1	
high	33	***0.006***	0.209(0.048–0.917)	***0.038***	***0.006***	0.206(0.047–0.902)	***0.036***

Abbreviations: *n.a.* Not application, *FIGO* the International Federation of Gynecology and Obstetrics; The bold number inside the table reflected *P* < 0.05

Finally, merging training cohort and validation cohort into overall cohort, the sample size was increased, and the similar analyses were also performed. The results demonstrated that low expression of TRIM62 was associated with the following factors: FIGO stage (*P* < 0.001), tumor size (*P* = 0.002), differentiation grade (*P* = 0.005), stromal invasion (*P* < 0.001), LVSI (*P* = 0.035), PLNM (*P* = 0.009), vaginal involvement (*P* = 0.044), recurrence (*P* < 0.001) and vital status at follow-up (*P* < 0.001) (Additional file 2: Table S2). There was no obvious relationship with age, pathologic types and parametrial infiltration (Additional file 2: Table S2). TRIM62 was an independent prognosis factor (OS: *P* = 0.003, DFS: *P* = 0.002) and its low expression associated with poor survival of CC (OS: *P* < 0.001, DFS: *P* < 0.001) was also verified in overall cohort (Fig. 2b, Additional file 2: Table S3 and S4).

Collectively, all these data demonstrated that TRIM62 was closely correlated with poor survival and might be used as a novel prognostic biomarker for early-stage CC.

### TRIM62 inhibits proliferation, migration and invasion of cervical cancer cells

According to the close association between low expression of TRIM62 and the tumor size as well as stromal invasion in early-stage CC patients (Table 1 and Additional file 2: Table S2), we further investigated the effects of TRIM62 on the proliferation, migration and invasion of CC cells. As was described above, TRIM62 protein was absent or markedly decreased in 62.5 % (5/8) of CC cell lines, including squamous and glandular CC cell lines (Fig. 1b). Therefore, we selected the most commonly used and low-expressed cell lines SiHa and HeLa as representatives for further studies of biological functions. TRIM62 was over-expressed in SiHa and HeLa cell lines by stable transfection of TRIM62 overexpression lentivirus, meanwhile the empty Lentivirus vectors were used as the negative control. The expression of TRIM62 in each cell line (SiHa-NC, SiHa-TRIM62, HeLa-NC, and HeLa-TRIM62) was identified by qRT-PCR and western blot (Additional file 3: Figure S2).

**Fig. 3:**
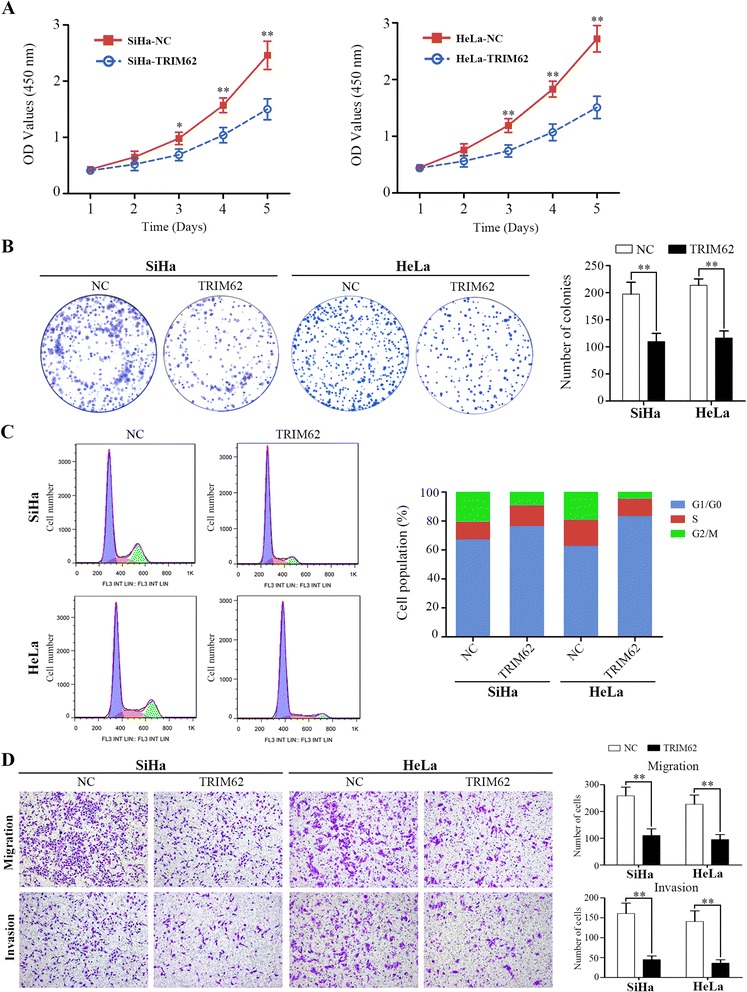
TRIM62 inhibits cervical cancer cells proliferation, migration and invasion in vitro. The stable cell lines SiHa-TRIM62 and HeLa-TRIM62 with up-regulated TRIM62 expression, as well as SiHa-NC and HeLa-NC (negative control) were used in the study. *, *P* < 0.05; **, *P* < 0.01. **a** CCK8 assays were used to detect the effect of TRIM62 on the viability of CC cells. **b** The effect of TRIM62 on colony formation of CC cells. **c** Flow cytometry assays were performed to analyze the effect of TRIM62 on the cell cycle of CC cells. **d** The transwell migration and transwell invasion assay were performed to investigate the effect of TRIM62 on the migration and invasion abilities of CC cell lines. Original magnification: ×100. **, *P* < 0.01

Firstly, CCK8 assay and colony formation assay were performed to assess the effect of TRIM62 on cell proliferation. SiHa-TRIM62 cells with higher expressed TRIM62 showed lower proliferation rate and less number of colonies compared with the control cells (SiHa-NC). Similarly, HeLa-TRIM62 cells also exhibited lower proliferation rate and less number of colonies than the control cells, HeLa-NC (Fig. 3a and b).

Secondly, we also performed cell cycle analysis to corroborate the effect of TRIM62 on proliferation of CC cells. The results showed that over-expressed TRIM62 in SiHa and HeLa cell lines demonstrated G1 cell cycle arrest, as was evidenced by the increased percentage of G1 and the reduced percentage of G2/M (Fig. 3c). These suggested that TRIM62 overexpression could block the cell cycle in G1 phase.

Additionally, in order to investigate the potential role of TRIM62 in modulating the migration and invasion ability of CC cells, we performed transwell migration and transwell invasion assays. Transwell migration assays revealed that overexpressing TRIM62 reduced the rate of migration in both SiHa-TRIM62 and HeLa-TRIM62 cells compared to the negative control cells (SiHa-NC or HeLa-NC), respectively (Fig. 3d). Similarly, as for the transwell invasion assays, both SiHa-TRIM62 and HeLa-TRIM62 showed reduced rate of invasion compared to the corresponding negative control SiHa-NC or HeLa-NC (Fig. 3d).

These data together suggested that overexpression of TRIM62 inhibited the proliferation, migration and invasion potentialities of CC cells.

### TRIM62 impedes growth and metastasis of cervical cancer in vivo

**Fig. 4:**
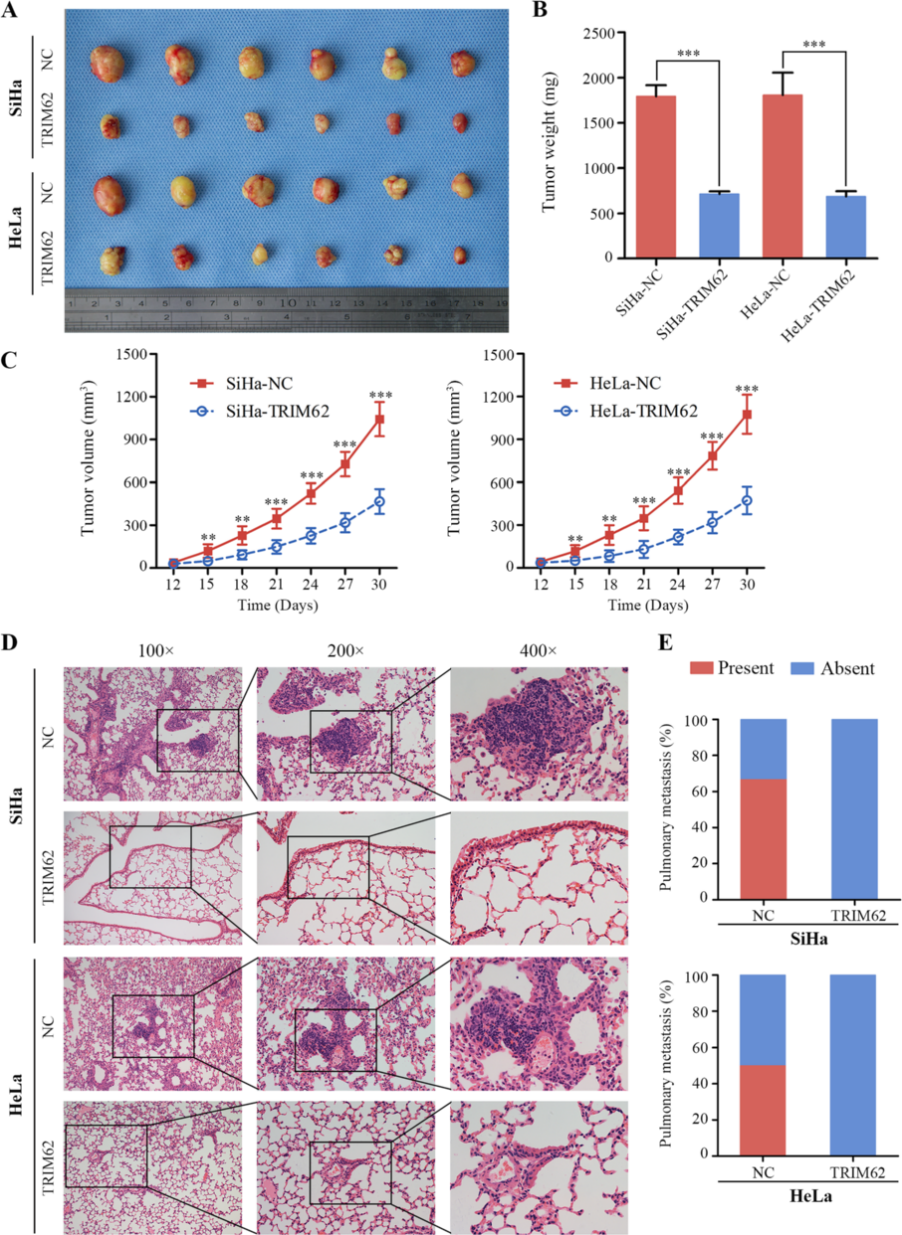
TRIM62 impedes growth and metastasis of cervical cancer in vivo*.* Effect of TRIM62 on tumor growth and metastasis were investigated in nude mouse xenograft model, which was built by injecting stable CC cells (SiHa-NC, SiHa-TRIM62, HeLa-NC and HeLa-TRIM62). **, *P* < 0.01; ***, *P* < 0.001. **a** A representative picture of tumors originated from nude mice, which had subcutaneously inoculated with stable CC cells as described above for 30 days. **b** Diagrams showed the calculation of the tumor weight of TRIM62 overexpressed groups (SiHa-TRIM62 and HeLa-TRIM62) compared with that of the corresponding control groups (SiHa-NC and HeLa-NC) (*n =* 6). **c** Subcutaneous tumor growth curves of each group were displayed (*n =* 6). **d** Typical H&E images of pulmonary metastatic foci in control groups and normal pulmonary tissues in TRIM62 overexpressed groups were shown, which indicating the metastatic capacity was decreased on TRIM62 overexpressed groups. Original magnification: ×100, ×200 and × 400. **e** The diagrams showed the percentages of mice with or without metastatic foci in the lungs (*n =* 6)

The effect of TRIM62 expression on tumor growth and metastasis were further verified by in vivo assays. We adopted subcutaneous xenograft tumor model and xenograft mouse metastatic model in the female BALB/c nude mice. After continuous monitoring 30 days, subcutaneous tumors in nude mice formed from the TRIM62-overexpressed group, SiHa-TRIM62 and HeLa-TRIM62, grew dramatically slower than those from their corresponding control group, SiHa-NC and HeLa-NC (Fig. 4a). The mean tumor weight and tumor volume of the TRIM62-overexpressed group, SiHa-TRIM62 and HeLa-TRIM62, were significantly smaller than their control group, SiHa-NC and HeLa-NC, respectively (Fig. 4b and c). To further determine the metastasis function of TRIM62 in vivo, xenograft mouse metastatic model were developed. After 6 weeks, lungs of the mice were removed and the tissues were made into H&E-stained paraffin sections. Microscopically, the incidences of pulmonary metastasis were decreased in the TRIM62-overexpressed groups (SiHa-TRIM62 and HeLa-TRIM62), compared to the corresponding control groups (SiHa-NC and HeLa-NC) (Fig. 4d and e).

Collectively, these results indicated that overexpressed TRIM62 played a role in inhibiting growth and metastasis of cervical cancer in vivo.

### TRIM62 suppresses epithelial-mesenchymal transition by inhibiting c-Jun/Slug signaling in cervical cancer

**Fig. 5:**
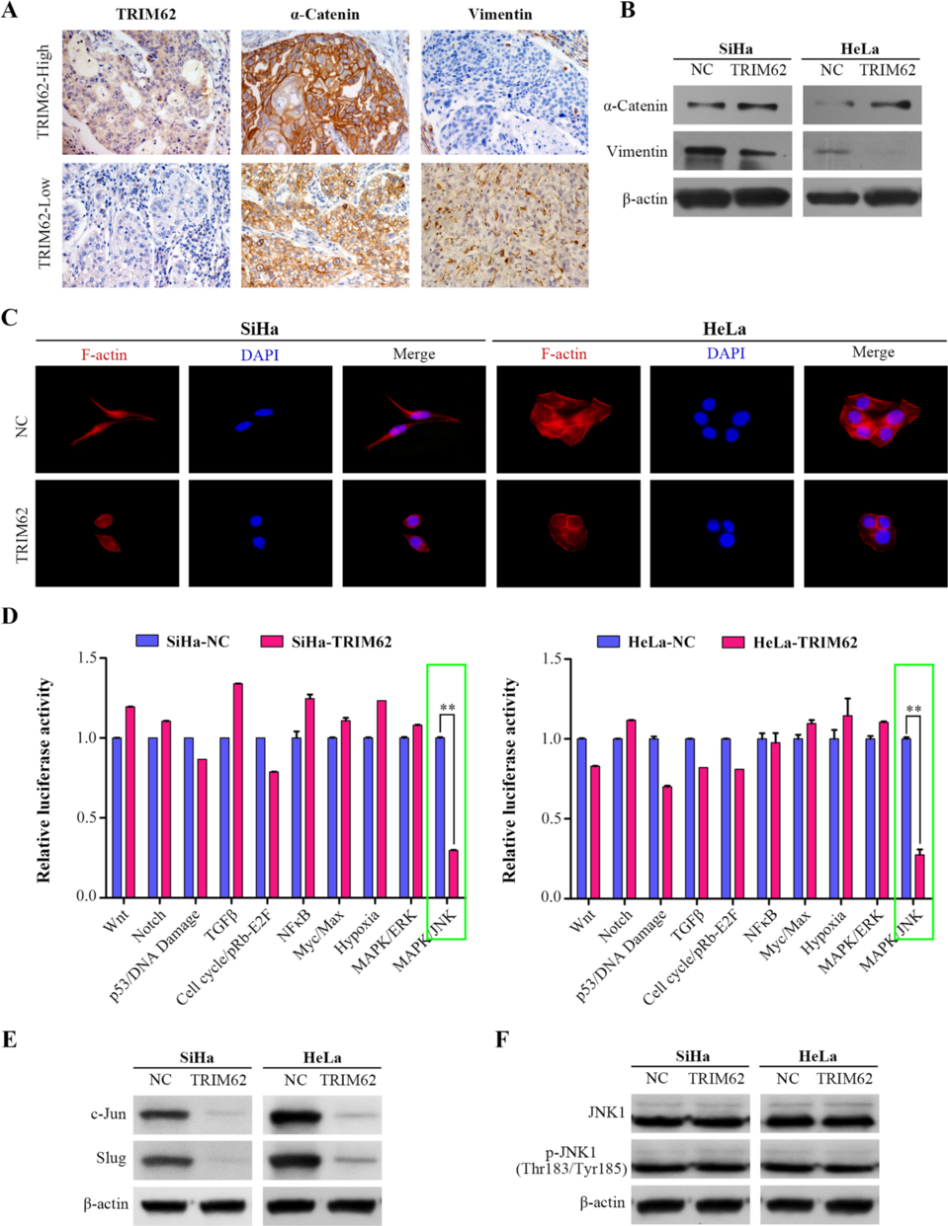
TRIM62 suppresses EMT by inhibiting c-Jun/Slug signaling in cervical cancer. **a** Representative IHC images of TRIM62 and EMT markers (α-Catenin and Vimentin). Original magnification: ×400. **b** Western blot analysis of α-Catenin and Vimentin expression in CC cells with or without TRIM62 overexpression. Beta-actin was used as a loading control. **c** Representative immunofluorescence images showed that TRIM62 affects the cellular morphology of CC cells. Cell nuclei were stained with DAPI (*blue*) and cytoskeleton (F-actin) were stained with rhodamine-phalloidin (*red*). Compared with the control cells respectively, SiHa-TRIM62 and HeLa-TRIM62 cells which overexpressed TRIM62 displayed cobblestone-like appearance with decreased F-actin fibers. Original magnification: ×400. **d** 10-Pathway Reporter Array showed the signaling changes in TRIM62-overexpressed cells compared with their corresponding controls. **, *P* < 0.01. **e** Protein expression levels of c-Jun and Slug detected by western blot were shown in indicated cells. **f** Protein levels of normal and phosphorylated form of JNK1 detected by western blot were shown in indicated cells

As confirmed above, both in vitro and in vivo experiments demonstrated overexpression of TRIM62 inhibits the metastasis of CC. Next we continued to identify its mechanism. It is reported that both in breast cancer and lung cancer TRIM62 was as a regulator of EMT [21, 23]. We therefore hypothesized TRIM62 was involved in the procedure of EMT in CC as well. Consequently, we firstly examined the association between TRIM62 and EMT markers (α-Catenin and Vimentin) expression in human cervical cancer by IHC (Fig. 5a). It discovered that TRIM62 expression was positively correlated with α-Catenin expression (*r* = 0.736, *P* = 0.001), whereas negatively correlated with Vimentin expression (*r* = -0.612, *P* = 0.003) in randomly selected cervical cancer sections (Additional file 2: Table S5). Moreover, expression of α-Catenin and Vimentin in overexpressed TRIM62 cells (SiHa-TRIM62 and HeLa-TRIM62) and their negative control cells were all detected by western blot as well. Consistent with the IHC results, expression level of α-Catenin was up-regulated in SiHa-TRIM62 and HeLa-TRIM62 cell lines, compared with their corresponding controls. On the contrary, expression of Vimentin was down-regulated after over-expression of TRIM62 in SiHa and HeLa cells (Fig. 5b). Furthermore, rhodamine-phalloidin fluorescent staining was used to track the influence of TRIM62 on cell morphology. SiHa-NC and HeLa-NC cells both exhibited elongated morphology with many long stretched F-actin fibers throughout the cytoplasm (mesenchymal phenotype-like). However, SiHa-TRIM62 and HeLa-TRIM62 cells displayed cobblestone-like appearance with decreased F-actin fibers (epithelial phenotype-like) (Fig. 5c). Taken together, these data demonstrated TRIM62 could suppress EMT in CC cells.

The potential mechanism underlying the suppressive effect of TRIM62 on EMT in CC cell lines was further investigated. To systemically screen out the potential signaling manipulated by TRIM62, a Cignal Finder Cancer 10-Pathway Reporter Array was adopted. The results indicated that MAPK/JNK signaling was dramatically suppressed after TRIM62 overexpression (Fig. 5d). However, MAPK/JNK signaling have been indicated 2 faces in cancer because of different AP-1 components [28, 29]. Based on the inhibitory role of TRIM62 in CC progression and MAPK/JNK signaling, we focused on the tumor-promoting role of MAPK/JNK signaling. As a classic proto-oncogene and a component of AP-1, c-Jun was found to be elevated in multiple cancer types, which shows a significant association with tumor invasion and metastasis [16, 30, 31]. Thus we speculated c-Jun was the main regulator of MAPK/JNK signaling after TRIM62 overexpression in CC cell lines. We next performed western blot to detect c-Jun. Notably, the expression of c-Jun was down-regulated after overexpressing TRIM62 (Fig. 5e). So how does the change in expression of c-Jun affect EMT? Several researches reported c-Jun could bind to the Slug promoter, which could result in an increase in expression of Slug and induction of EMT [13, 17]. Then we detected the expression of Slug, and found it was also attenuated in TRIM62-overexpression group (Fig. 5e). To clarify how the expression of c-Jun was inhibited by TRIM62, we also measured JNK1, the upstream protein of c-Jun in MAPK/JNK signaling [32, 33]. We detected its normal and phosphorylated forms. Results demonstrated both normal and phosphorylated forms of JNK1 were not affected by TRIM62 (Fig. 5f). These results indicated the inhibitory function of TRIM62 on MAPK/JNK signaling was through suppressing the expression of c-Jun.

With all these results taken together, it is concluded that TRIM62 could suppress EMT by inhibiting c-Jun/Slug signaling in CC.

### Rescue of c-Jun abrogates inhibition of TRIM62 on cell migration and invasion

**Fig. 6:**
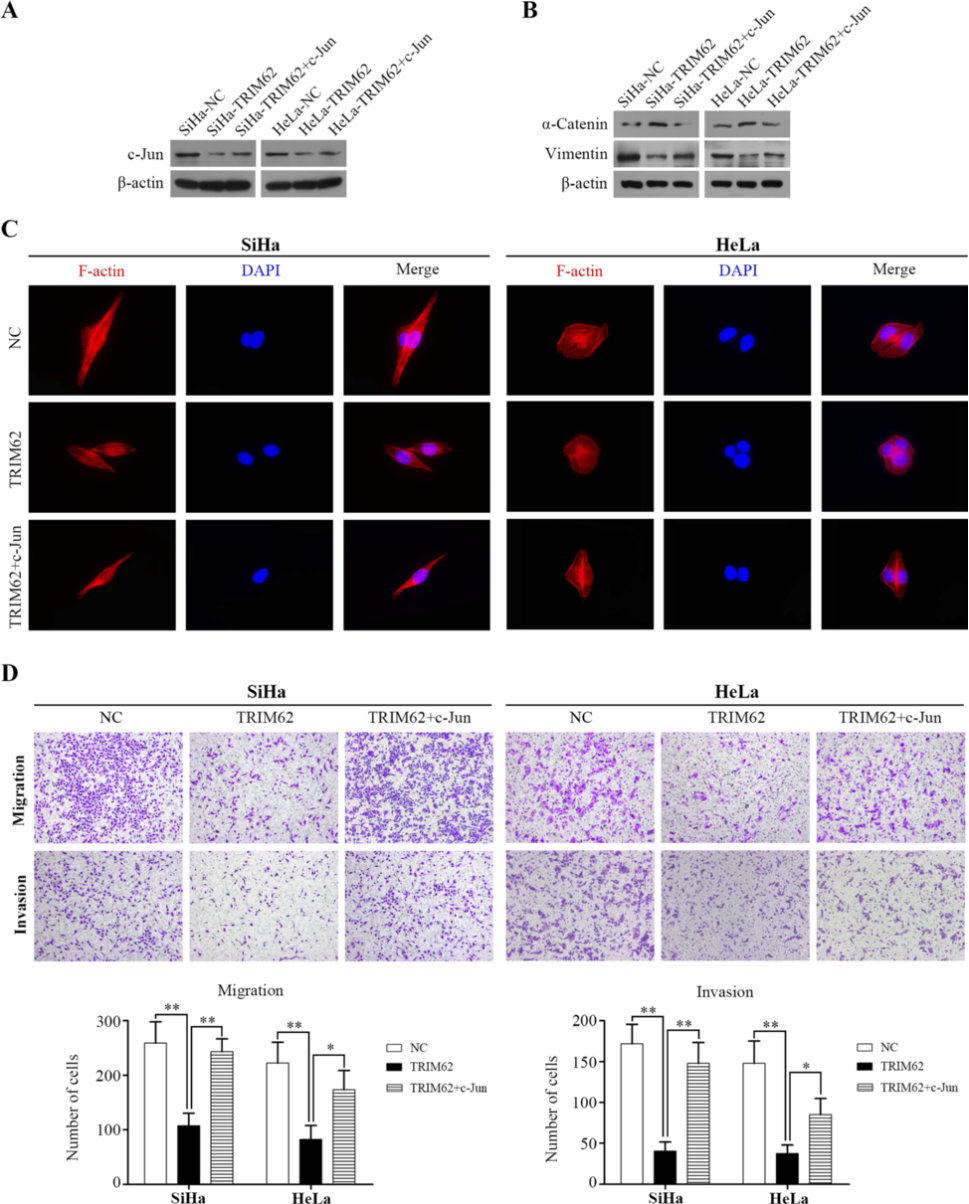
Rescue of c-Jun abrogates inhibition of TRIM62 on cell migration and invasion. **a** Expression of c-Jun protein in SiHa-NC, SiHa-TRIM62, SiHa-TRIM62 + c-Jun, HeLa-NC, HeLa-TRIM62 and HeLa-TRIM62 + c-Jun cells was detected by western blot. **b** Western blot detected the expression level of α-Catenin and Vimentin in TRIM62-overexpressed cells after overexpression of c-Jun. **c** Representative immunofluorescence images showed that epithelial phenotype-like morphology of TRIM62-overexpressed cells changed back to mesenchymal phenotype-like morphology after rescuing the expression of c-Jun. Cell nuclei were stained with DAPI (blue) and cytoskeleton (F-actin) were stained with rhodamine-phalloidin (*red*). Original magnification: ×400. **d** The transwell migration and transwell invasion assays were performed to investigate the effect of c-Jun overexpression on the migration and invasion abilities of TRIM62-overexpressed CC cell lines. The inhibited migration and invasion by gain of TRIM62 were significantly abrogated after overexpression of c-Jun in TRIM62-overexpressed cells. Original magnification: ×100. *, P < 0.05; **, P < 0.01

Having demonstrated TRIM62 could suppress EMT by inhibiting c-Jun/Slug signaling, we wanted to further clarify whether the inhibition effect of TRIM62 on cell invasion and migration was through suppressing c-Jun/Slug signaling-mediated EMT. We transfected c-Jun overexpression lentivirus into TRIM62-overexpressed cells, and the rescue of c-Jun expression in these cells was validated by western blot (Fig. 6a). Firstly, the expression level of α-Catenin was significantly decreased after overexpression of c-Jun in TRIM62-overexpressed cells. While the expression level of Vimentin was significantly increased after overexpression of c-Jun in TRIM62-overexpressed cells (Fig. 6b). Then cell function assays were performed. We detected cell morphology with rhodamine-phalloidin fluorescent staining. It demonstrated that epithelial phenotype-like morphology of TRIM62-overexpressed cells changed back to mesenchymal phenotype-like morphology after rescuing the expression of c-Jun (Fig. 6c). Morever, transwell migration and transwell invasion assays were further adopted. Results showed the inhibited migration and invasion by gain of TRIM62 were significantly abrogated after overexpression of c-Jun in TRIM62-overexpressed cells (Fig. 6d). All these results suggested rescue of c-Jun could abrogate inhibition of TRIM62 on cell migration and invasion.

### TRIM62 regulates the expression of CyclinD1 and P27 via targeting c-Jun in cervical cancer

We next tried to explore the mechanism of the inhibitory role of TRIM62 on proliferation in CC. As detailed above, TRIM62 overexpression could block the cell cycle in G1 phase. Thus we detected the expression of cell cycle related protein CyclinD1, a key regulator of the G1 to S phase transition, and P27, a negative regulator of the cell cycle by inhibiting cell progression to S phase. Western blot analysis revealed that CyclinD1 was down-regulated, whereas P27 was up-regulated in TRIM62 overexpressed cells compared with the negative control cells (Additional file 4: Figure S3A). It implied that TRIM62 overexpression resulted in lower expression of CyclinD1 with higher expression of P27. The results suggested that the inhibitory role of TRIM62 in CC proliferation might through decreasing the expression of CyclinD1 and increasing the P27 expression, which could lead to blocking the cell cycle in G1 phase. But how could TRIM62 alter the expression of CyclinD1 and P27? As confirmed above, TRIM62 could suppress MAPK/JNK signaling by down-regulating c-Jun. In addition, MAPK/JNK signaling also has been reported to participate in cell proliferation and tumor growth, and CyclinD1 and P27 could be regulated by c-Jun [34, 35]. So we hypothesized that these changes caused by TRIM62 were through targeting c-Jun. We detected the expression level of CyclinD1 and P27 before and after c-Jun overexpressed in TRIM62-overexpressed cells by western blot. Results displayed the expression level of CyclinD1 was significantly increased after overexpression of c-Jun. While the expression level of P27 was significantly decreased after c-Jun overexpression (Additional file 4: Figure S3B). All these results suggested TRIM62 regulated the expression of cell cycle regulator CyclinD1 and P27 via targeting c-Jun. This might be the potential molecular mechanism that leads to reduced cell proliferation after TRIM62 overexpression.

## Discussion

To date, approximately 100 human *TRIM* genes have been identified and their alterations are associated with diverse pathological conditions, such as developmental disorders, neurodegenerative diseases, viral infections and cancer [36, 37]. Previous evidence indicates that whether TRIMs function as oncogenes or tumor suppressor genes is context dependent. For example, TRIM25 is overexpressed in ovarian cancer, but is down-regulated in endometrial cancer [36]. Here we explored the role of TRIM62 in CC and identified that TRIM62 was not only frequently down-regulated in both CC tissues and cell lines, but also acted as an independent predictor of both OS and DFS in early-stage CC. Moreover, it significantly inhibited proliferation and metastasis of cervical cancer both in vitro and in vivo, which suggested that TRIM62 played as a tumor suppressor in CC. In addition, mechanism research indicated TRIM62 could suppress tumor metastasis via inhibiting c-Jun/Slug signaling-mediated EMT, and its inhibitory role in tumor proliferation might be through regulating cell cycle related proteins CyclinD1 and P27 by targeting c-Jun in CC. To the best of our knowledge, this is the first study exploring the relationship between TRIM62 and CC progression. However, there still exist several potential problems to be further discussed and explored.

First of all, as for the correlation analyses between TRIM62 expression and clinicopathologic characteristics, we noticed several different results between the training cohort and the validation cohort. PLNM was one of the different results, which was considered as a critical factor for determining the individualized treatment and prognosis of cervical cancer, particularly in early-stage CC [38]. Limited by the sample size of validation cohort and the lack of multicenter statistics, we cannot deny the correlation of TRIM62 with PLNM. In addition, it has been well documented that the most common pattern of metastasis of CC is direct extension. This study showed that TRIM62 low-expression was significantly associated with stromal invasion, which was an indicator of direct extension. As a result, we supposed down-regulation of TRIM62 might take part in the metastasis of early-stage CC mainly through accelerating direct extension. Moreover, this result also supported the functional research that demonstrated the inhibition effect of TRIM62 on tumor metastasis.

In mechanism studies, we principally focused on clarifying how TRIM62 suppressed CC metastasis, as local invasion and distant metastasis rather than cell proliferation itself are the cause of 90 % of human cancer deaths [6, 14, 39]. Previous studies indicated TRIM62 was a suppressor of EMT in breast cancer and lung cancer [21, 23]. In this study we obtained the similar results that TRIM62 could inhibit EMT in CC. However, different from the results found in breast cancer that TRIM62 acted as a master regulator on EMT by blocking TGF-β signaling [21], the results of Multi-pathway reporter array showed that in CC TGF-β signaling was not significantly affected by TRIM62. We identified that TRIM62 could suppress MAPK/JNK signaling by down-regulating c-Jun and inhibit EMT via targeting c-Jun/Slug signaling in CC. Additionally, we for the first time explored the relationship between TRIM62 and cell proliferation, and found that TRIM62 overexpression could block the cell cycle in G1 phase. As TRIM62 could suppress MAPK/JNK signaling by down-regulating c-Jun and accumulating evidences demonstrated MAPK/JNK signaling also participated in cell proliferation and tumor growth, we next tried to clarify the possible link between MAPK/JNK signaling and TRIM62 in the aspect of CC proliferation. Our results suggested that TRIM62 regulated the expression of cell cycle regulator CyclinD1 and P27 via targeting c-Jun in CC, which might be the molecular mechanism of TRIM62 inhibiting proliferation in CC. We observed that overexpressed c-Jun in TRIM62 overexpression CC cells resulted in up-regulated CyclinD1 expression and down-regulated P27 expression. It is reported that c-Jun was able to activate the promoter of cyclinD1 [34], and CyclinD1 could act as a negative regulator of P27 [40]. So we speculated c-Jun might up-regulate CyclinD1 expression by activating its promoter, consequently down-regulating P27 expression. However, Khattar et al also indicated that c-Jun could combine with c-Fos forming one kind of AP-1 complexes (c-Jun/c-Fos) thereby resulting in P27 down-regualtion [35]. Thus the underlying mechanism of down-regulated P27 by c-Jun needs further investigation. Furthermore, our results suggested that the function of TRIM62 suppressing both CC proliferation and metastasis was through down-regulating c-Jun expression. But how did TRIM62 attenuate the expression of c-Jun? Multiple E3 ligases such as COP1, ITCH and FBW7 have been reported to mediate the ubiquitination and proteasomal degradation of c-Jun [41–43]. Identification of additional ubiquitin ligases that regulate the ubiquitination of c-Jun will provide a better understanding of the regulation of c-Jun. As a RING finger E3 ubiquitin ligase [18, 19], TRIM62 has been associated with the formation and architecture of large protein complexes. Recent studies also showed that TRIM62 can directly bind to SMAD3 or CARD9 thereby promoting the ubiquitination and proteasome-mediated degradation of those two proteins [21, 44]. Therefore, we supposed that TRIM62 might bind to c-Jun and down-regulate the expression of c-Jun by facilitating the ubiquitination and proteasomal degradation of c-Jun. This remains to be further explored.

Our results demonstrated that the expression of TRIM62 were markedly down-regulated at both mRNA and protein level. So it seems that down-regulated expression of TRIM62 might undergo reduced gene transcription in CC. Genomic aberrations and epigenetic regulation are two main methods, which could down-regulate the expression of potential tumor suppressor genes in tumor tissues [45, 46]. Lott et al discovered that *TRIM62* gene is mutated and deleted in breast cancer. However, they analysis both 14 breast cancer cell lines and 20 tumor samples and did not found promoter methylation existing in any of the samples [20]. Thus we speculated that down-regulated TRIM62 in CC might probably be caused by the mutation and deletion of *TRIM62* gene. This needs to be further investigated.

## Conclusions

In conclusion, our data suggest that TRIM62 could serve as a novel prognostic indicator for early-stage CC patients. In addition, in vitro and in vivo experiments validated the inhibitory role of TRIM62 in CC growth and metastasis. Meanwhile, we further found the inhibitory role of TRIM62 in metastasis was through suppressing c-Jun/Slug signaling-mediated EMT. The inhibitory role of TRIM62 on tumor proliferation might be through regulating cell cycle related proteins CyclinD1 and P27 by targeting c-Jun in CC. Therefore, we suppose strategies designed to up-regulate TRIM62 may provide a promising method to alleviate CC progression.

## Additional files

Additional file 1: Figure S1. Flow diagram of CC patients enrolled in this study. (TIF 196 kb)
Additional file 2: Supplementary tables of this study. (DOCX 39 kb)
Additional file 3: Figure S2. Transfection related results. The expression level of TRIM62 was normalized to β-actin. The error bars represent standard deviation values calculated from three parallel experiments. *, *P* < 0.05; **, *P* < 0.01. (A) Representative light-field and fluorescence images of stable CC cells (SiHa-NC, SiHa-TRIM62, HeLa-NC and HeLa-TRIM62). The green fluorescence protein was detected in an inverted fluorescence microscope DMI4000B (Leica, Wetzlar, Germany). Original magnification: ×100. (B) Expression of TRIM62 protein in SiHa-Blank, HeLa-Blank and stable NC-or TRIM62-transduced SiHa and HeLa cells. (C) Relative mRNA expression of TRIM62 in SiHa-Blank, HeLa-Blank and stable NC-or TRIM62-transduced SiHa and HeLa cells was detected by qRT-PCR. The expression of TRIM62 in CC cells with TRIM62 over-expressed (SiHa-TRIM62 and HeLa-TRIM62) were dramatically increased than that in their corresponding negative control cells (SiHa-NC and HeLa-NC), at both mRNA and protein levels. Simultaneously, the expression of TRIM62 between SiHa-Blank and SiHa-NC or between HeLa-Blank and HeLa-NC showed no statistical significance. (TIF 1059 kb)
Additional file 4: Figure S3. The expression level of cell cycle related proteins CyclinD1 and P27. Beta-actin was used as a loading control. (A) Western blot detected the expression level of CyclinD1 and P27 in CC cells with or without TRIM62 overexpression. (B) Expression level of CyclinD1 and P27 in SiHa-NC, SiHa-TRIM62, SiHa-TRIM62 + c-Jun, HeLa-NC, HeLa-TRIM62 and HeLa-TRIM62 + c-Jun cells were detected by western blot. (TIF 372 kb)

## Abbreviations

AC: Adenocarcinoma
ANT: Adjacent noncancerous tissue
CC: Cervical cancer
DFS: Disease-free survival
EMT: Epithelial mesenchymal transition
FIGO: The International Federation of Gynecology and Obstetrics
mRNA: messenger RNA
NC: Negative control
NCT: Normal cervical tissue
OS: Overall survival
PLNM: Pelvic lymph node metastasis
qRT-PCR: Quantitative Real-time PCR
SCC: Squamous cell carcinoma
TRIM62: Tripartite motif containing 62
