# Supplementary figures and images for: Tripartite motif containing 62 is a novel prognostic marker and suppresses tumor metastasis via c-Jun/Slug signaling-mediated epithelial-mesenchymal transition in cervical cancer
Source: J Exp Clin Cancer Res. 2016 Oct 28;35:170. doi: 10.1186/s13046-016-0445-5 (PMC5084416; doi:10.1186/s13046-016-0445-5)

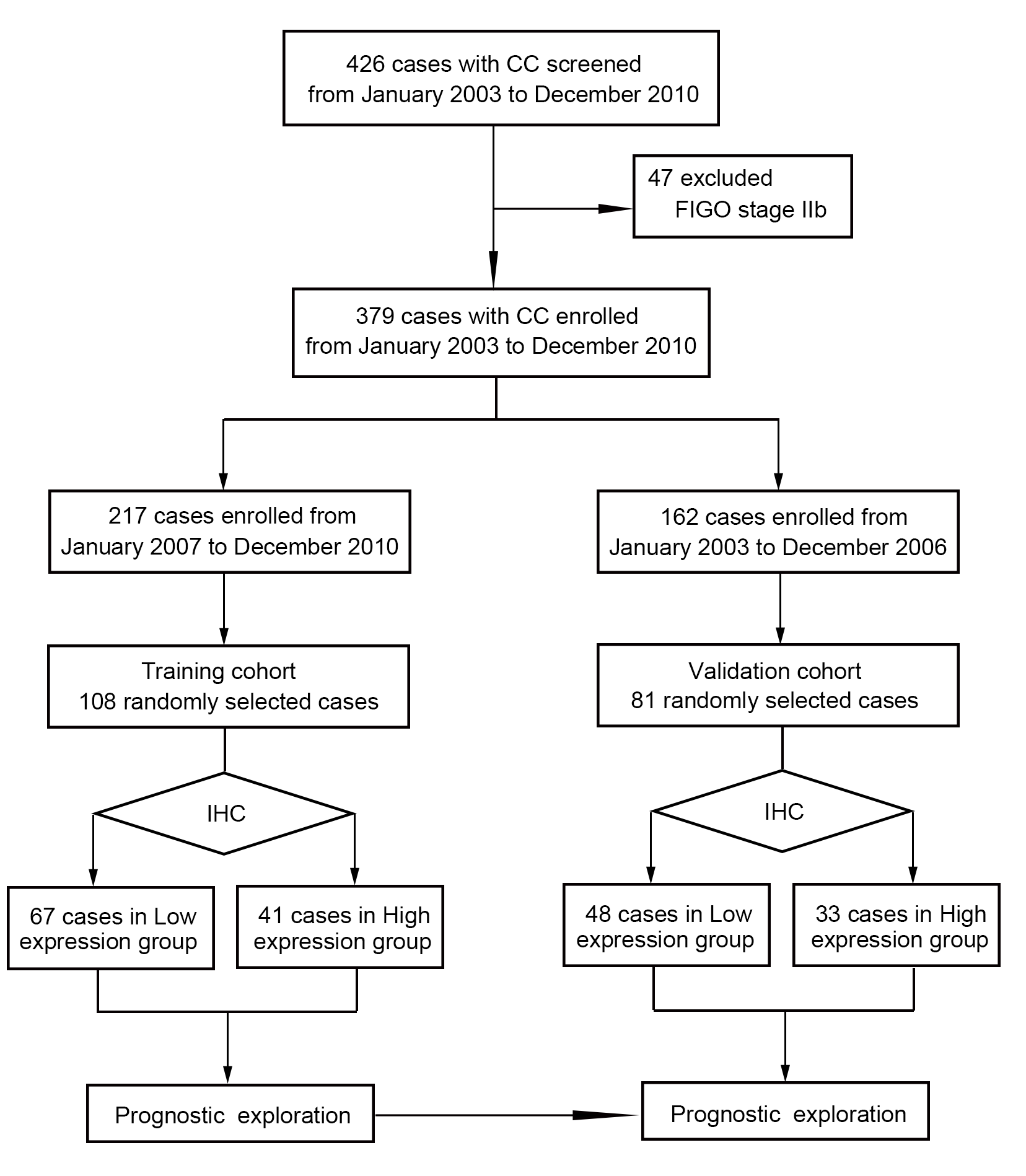

Supplement: Additional file 1: Figure S1. — Flow diagram of CC patients enrolled in this study. (TIF 196 kb) [file 13046_2016_445_MOESM1_ESM.tif]

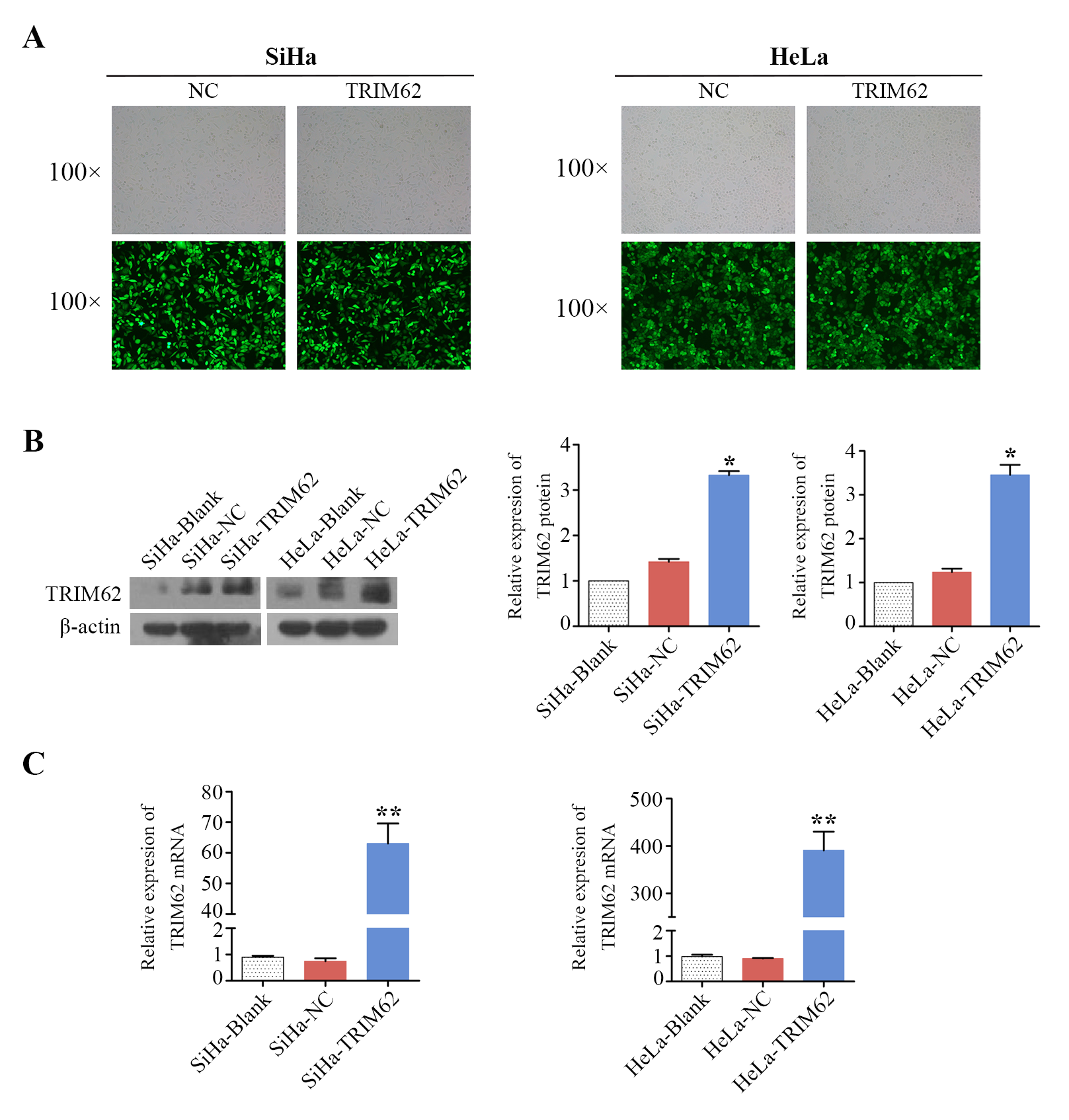

Supplement: Additional file 3: Figure S2. — Transfection related results. The expression level of TRIM62 was normalized to β-actin. The error bars represent standard deviation values calculated from three parallel experiments. *, P < 0.05; **, P < 0.01. (A) Representative light-field and fluorescence images of stable CC cells (SiHa-NC, SiHa-TRIM62, HeLa-NC and HeLa-TRIM62). The green fluorescence protein was detected in an inverted fluorescence microscope DMI4000B (Leica, Wetzlar, Germany). Original magnification: ×100. (B) Expression of TRIM62 protein in SiHa-Blank, HeLa-Blank and stable NC-or TRIM62-transduced SiHa and HeLa cells. (C) Relative mRNA expression of TRIM62 in SiHa-Blank, HeLa-Blank and stable NC-or TRIM62-transduced SiHa and HeLa cells was detected by qRT-PCR. The expression of TRIM62 in CC cells with TRIM62 over-expressed (SiHa-TRIM62 and HeLa-TRIM62) were dramatically increased than that in their corresponding negative control cells (SiHa-NC and HeLa-NC), at both mRNA and protein levels. Simultaneously, the expression of TRIM62 between SiHa-Blank and SiHa-NC or between HeLa-Blank and HeLa-NC showed no statistical significance. (TIF 1059 kb) [file 13046_2016_445_MOESM3_ESM.tif]

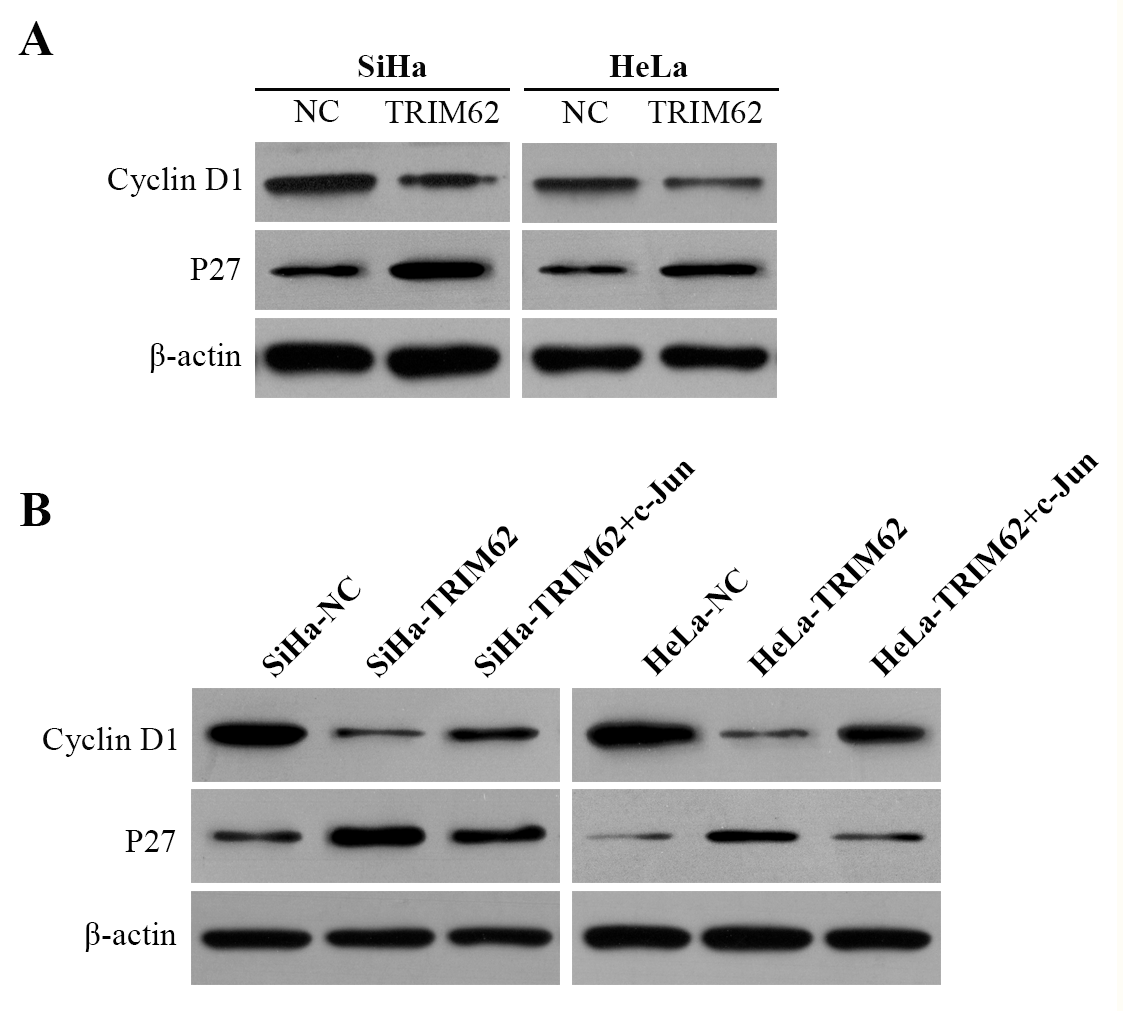

Supplement: Additional file 4: Figure S3. — The expression level of cell cycle related proteins CyclinD1 and P27. Beta-actin was used as a loading control. (A) Western blot detected the expression level of CyclinD1 and P27 in CC cells with or without TRIM62 overexpression. (B) Expression level of CyclinD1 and P27 in SiHa-NC, SiHa-TRIM62, SiHa-TRIM62 + c-Jun, HeLa-NC, HeLa-TRIM62 and HeLa-TRIM62 + c-Jun cells were detected by western blot. (TIF 372 kb) [file 13046_2016_445_MOESM4_ESM.tif]
