# Supplementary material for: Tripartite motif containing 62 is a novel prognostic marker and suppresses tumor metastasis via c-Jun/Slug signaling-mediated epithelial-mesenchymal transition in cervical cancer
Source: J Exp Clin Cancer Res. 2016 Oct 28;35:170. doi: 10.1186/s13046-016-0445-5 (PMC5084416; doi:10.1186/s13046-016-0445-5)
Supplement: Additional file 2: — Supplementary tables of this study. (DOCX 39 kb) [file 13046_2016_445_MOESM2_ESM.docx]

**Supplementary tables**

**Table S1** Clinicopathologc Characteristics of Patients in Training Cohort and Validation Cohort

| **Clinicopathologic Variable** | **Counts** | |  |
| --- | --- | --- | --- |
|  | **Training cohort** | **Validation cohort** | ***P*-value** |
| **Age(years)** |  |  |  |
| ≦42 | 61(56.5) | 44(54.3) | 0.767 |
| ＞42 | 47(43.5) | 37(45.7) |  |
| **FIGO stage** |  |  |  |
| Ia2 | 9(8.3) | 11(13.6) | 0.804 |
| Ib1 | 56(51.9) | 39(48.1) |  |
| Ib2 | 18(16.7) | 14(17.3) |  |
| IIa1 | 12(11.1) | 7(8.6) |  |
| IIa2 | 13(12.0) | 10(12.3) |  |
| **Tumor size(cm)** |  |  |  |
| ≦4 | 74(68.5) | 54(66.7) | 0.788 |
| ＞4 | 34(31.5) | 27(33.3) |  |
| **Pathologic types** |  |  |  |
| Squamous cell carcinoma | 68(63.0) | 53(65.4) | 0.726 |
| Adenocarcinoma | 40(37.0) | 28(34.6) |  |
| **Differentiation grade** |  |  |  |
| Well | 9(8.3) | 7(8.6) | 0.997 |
| Moderate | 48(44.4) | 36(44.4) |  |
| Poor | 51(47.3) | 38(46.9) |  |
| **Stromal invasion** |  |  |  |
| ＜1/2 | 61(56.5) | 50(61.7) | 0.468 |
| ≧1/2 | 47(43.5) | 31(38.3) |  |
| **Lymphovascular space invasion** |  |  |  |
| Yes | 14(13.0) | 11(13.6) | 0.901 |
| No | 94(87.0) | 70(86.4) |  |
| **Pelviclymph node metastasis** |  |  |  |
| Yes | 18(16.7) | 14(17.3) | 0.911 |
| No | 90(83.3) | 67(82.7) |  |
| **Vaginal involvement** |  |  |  |
| Yes | 4(3.7) | 3(3.7) | 1.000^a^ |
| No | 104(96.3) | 78(96.3) |  |
| **Parametrial infiltration** |  |  |  |
| Yes | 3(2.8) | 2(2.5) | 1.000^a^ |
| No | 105(97.2) | 79(97.5) |  |
| **Recurrence** |  |  |  |
| Yes | 24(22.2) | 20(24.7) | 0.691 |
| No | 84(77.8) | 61(75.3) |  |
| **Vital status at follow-up** |  |  |  |
| Alive | 86(79.6) | 61(75.3) | 0.480 |
| Dead | 22(20.4) | 20(24.7) |  |

Abbreviations: FIGO, the International Federation of Gynecology and Obstetrics;

^a^ P-value from Fisher’s exact test.

**Table S2** Correlation between TRIM62 Expression and Clinicopathologic Characteristics of Early-stage CC in Overall Cohort

| **Clinicopathologic Variable** | **Total** | **TRIM62** | | ***P*-value** |
| --- | --- | --- | --- | --- |
|  |  | **low** | **High** |  |
| **Age(years)** |  |  |  |  |
| ≦42 | 105 | 66 | 39 | 0.527 |
| ＞42 | 84 | 49 | 35 |  |
| **FIGO stage** |  |  |  |  |
| Ia2 | 20 | 4 | 16 | ***＜0.001*** |
| Ib1 | 95 | 56 | 39 |  |
| Ib2 | 32 | 21 | 11 |  |
| IIa1 | 19 | 14 | 5 |  |
| IIa2 | 23 | 20 | 3 |  |
| **Tumor size(cm)** |  |  |  |  |
| ≦4 | 128 | 68 | 60 | ***0.002*** |
| ＞4 | 61 | 47 | 14 |  |
| **Pathologic types** |  |  |  |  |
| Squamous cell carcinoma | 121 | 73 | 48 | 0.846 |
| Adenocarcinoma | 68 | 42 | 26 |  |
| **Differentiation grade** |  |  |  |  |
| Well | 16 | 7 | 9 | ***0.005*** |
| Moderate | 84 | 43 | 41 |  |
| Poor | 89 | 65 | 24 |  |
| **Stromal invasion** |  |  |  |  |
| ＜1/2 | 111 | 56 | 55 | ***＜0.001*** |
| ≧1/2 | 78 | 59 | 19 |  |
| **Lymphovascular space invasion** |  |  |  |  |
| Yes | 25 | 20 | 5 | ***0.035*** |
| No | 164 | 95 | 69 |  |
| **Pelvic-lymph node metastasis** |  |  |  |  |
| Yes | 32 | 26 | 6 | ***0.009*** |
| No | 157 | 89 | 68 |  |
| **Vaginal involvement** |  |  |  |  |
| Yes | 7 | 7 | 0 | ***0.044^a^*** |
| No | 182 | 108 | 74 |  |
| **Parametrial infiltration** |  |  |  |  |
| Yes | 5 | 5 | 0 | 0.158^a^ |
| No | 184 | 110 | 74 |  |
| **Recurrence** |  |  |  |  |
| Yes | 44 | 40 | 4 | ***＜0.001*** |
| No | 145 | 75 | 70 |  |
| **Vital status at follow-up** |  |  |  |  |
| Alive | 147 | 77 | 70 | ***＜0.001*** |
| Dead | 42 | 38 | 4 |  |

Abbreviations: FIGO, the International Federation of Gynecology and Obstetrics;

^a^ P-value from Fisher’s exact test.

**Table S3** Univariate and Multivariate Analyses of Factors Associated with Overall Survival in Overall Cohort

| **Clinicopathologic Variable** | **Total** | **Univariate analysis** | |  | **Multivariate analysis** | |
| --- | --- | --- | --- | --- | --- | --- |
|  |  | **HR(95%CI)** | ***P*-value** |  | **HR(95%CI)** | ***P*-value** |
| **Age(years)** |  |  |  |  |  |  |
| ≦42 | 105 | 1 |  |  |  |  |
| ＞42 | 84 | 0.909(0.493-1.675) | 0.760 |  | n.a. | n.a. |
| **FIGO stage** |  |  |  |  |  |  |
| Ia2 | 20 | 1 |  |  | 1 |  |
| Ib1 | 95 |  |  |  |  |  |
| Ib2 | 32 |  |  |  |  |  |
| IIa1 | 19 |  |  |  |  |  |
| IIa2 | 23 | 1.894(1.499-2.394) | ***＜0.001*** |  | 1.919(1.411-2.609) | ***＜0.001*** |
| **Tumor size(cm)** |  |  |  |  |  |  |
| ≦4 | 128 | 1 |  |  | 1 |  |
| ＞4 | 61 | 2.131(1.163-3.906) | ***0.014*** |  | 0.597(0.280-1.272) | 0.181 |
| **Pathologic types** |  |  |  |  |  |  |
| Squamous cell carcinoma | 121 | 1 |  |  |  |  |
| Adenocarcinoma | 68 | 1.116(0.599-2.081) | 0.729 |  | n.a. | n.a. |
| **Differentiation grade** |  |  |  |  |  |  |
| Well | 16 | 1 |  |  | 1 |  |
| Moderate | 84 |  |  |  |  |  |
| Poor | 89 | 1.729(1.021-2.928) | ***0.042*** |  | 1.701(0.997-2.902) | 0.051 |
| **Stromal invasion** |  |  |  |  |  |  |
| ＜1/2 | 111 | 1 |  |  | 1 |  |
| ≧1/2 | 78 | 2.992(1.591-5.628) | ***0.001*** |  | 1.868(0.921-3.789) | 0.083 |
| **Lymphovascular space invasion** |  |  |  |  |  |  |
| Yes | 25 | 1 |  |  | 1 |  |
| No | 164 | 2.228(1.094-4.534) | ***0.027*** |  | 0.594(0.236-1.493) | 0.268 |
| **Pelvic-lymph node metastasis** |  |  |  |  |  |  |
| Yes | 32 | 1 |  |  | 1 |  |
| No | 157 | 2.865(1.508-5.445) | ***0.001*** |  | 2.007(0.913-4.415) | 0.083 |
| **Vaginal involvement** |  |  |  |  |  |  |
| Yes | 7 | 1 |  |  |  |  |
| No | 182 | 1.312(0.317-5.429) | 0.708 |  | n.a. | n.a. |
| **Parametrial infiltration** |  |  |  |  |  |  |
| Yes | 5 | 1 |  |  |  |  |
| No | 184 | 2.498(0.603-10.338) | 0.207 |  | n.a. | n.a. |
| **TRIM62** |  |  |  |  |  |  |
| low | 115 | 1 |  |  | 1 |  |
| high | 74 | 0.135(0.048-0.379) | ***＜0.001*** |  | 0.211(0.075-0.594) | ***0.003*** |

Abbreviations: n.a., Not application; FIGO, the International Federation of Gynecology and Obstetrics;

**Table S4** Univariate and Multivariate Analyses of Factors Associated with Disease-Free Survival in Overall Cohort

| **Clinicopathologic Variable** | **Total** | **Univariate analysis** | |  | **Multivariate analysis** | |
| --- | --- | --- | --- | --- | --- | --- |
|  |  | **HR(95%CI)** | ***P*-value** |  | **HR(95%CI)** | ***P*-value** |
| **Age(years)** |  |  |  |  |  |  |
| ≦42 | 105 | 1 |  |  |  |  |
| ＞42 | 84 | 0.908(0.500-1.650) | 0.752 |  | n.a. | n.a. |
| **FIGO stage** |  |  |  |  |  |  |
| Ia2 | 20 | 1 |  |  | 1 |  |
| Ib1 | 95 |  |  |  |  |  |
| Ib2 | 32 |  |  |  |  |  |
| IIa1 | 19 |  |  |  |  |  |
| IIa2 | 23 | 1.876(1.492-2.358) | ***＜0.001*** |  | 1.920(1.423-2.590) | ***＜0.001*** |
| **Tumor size(cm)** |  |  |  |  |  |  |
| ≦4 | 128 | 1 |  |  | 1 |  |
| ＞4 | 61 | 1.956(1.081-3.541) | ***0.027*** |  | 0.560(0.269-1.167) | 0.122 |
| **Pathologic types** |  |  |  |  |  |  |
| Squamous cell carcinoma | 121 | 1 |  |  |  |  |
| Adenocarcinoma | 68 | 1.043(0.564-1.928) | 0.894 |  | n.a. | n.a. |
| **Differentiation grade** |  |  |  |  |  |  |
| Well | 16 | 1 |  |  | 1 |  |
| Moderate | 84 |  |  |  |  |  |
| Poor | 89 | 1.807(1.072-3.043) | ***0.026*** |  | 1.702(1.005-2.882) | ***0.048*** |
| **Stromal invasion** |  |  |  |  |  |  |
| ＜1/2 | 111 | 1 |  |  | 1 |  |
| ≧1/2 | 78 | 2.866(1.550-5.299) | ***0.001*** |  | 1.823(0.923-3.600) | 0.084 |
| **Lymphovascular space invasion** |  |  |  |  |  |  |
| Yes | 25 | 1 |  |  | 1 |  |
| No | 164 | 2.311(1.168-4.575) | ***0.016*** |  | 0.656(0.275-1.565) | 0.342 |
| **Pelvic-lymph node metastasis** |  |  |  |  |  |  |
| Yes | 32 | 1 |  |  | 1 |  |
| No | 157 | 3.104(1.236-7.791) | ***0.016*** |  | 1.841(0.864-3.924) | 0.114 |
| **Vaginal involvement** |  |  |  |  |  |  |
| Yes | 7 | 1 |  |  |  |  |
| No | 182 | 1.349(0.326-5.576) | 0.679 |  | n.a. | n.a. |
| **Parametrial infiltration** |  |  |  |  |  |  |
| Yes | 5 | 1 |  |  |  |  |
| No | 184 | 2.288(0.554-9.456) | 0.253 |  | n.a. | n.a. |
| **TRIM62** |  |  |  |  |  |  |
| low | 115 | 1 |  |  | 1 |  |
| high | 74 | 0.127(0.045-0.356) | ***＜0.001*** |  | 0.196(0.070-0.551) | ***0.002*** |

Abbreviations: n.a., Not application; FIGO, the International Federation of Gynecology and Obstetrics;

**Table S5** TRIM62 is positively correlated with α-Catenin and negatively correlated with Vimentin

| Protein |  | α-Catenin | | | |  | Vimentin | | | |
| --- | --- | --- | --- | --- | --- | --- | --- | --- | --- | --- |
|  |  | Low | High | *r* | *P* |  | Low | High | *r* | *P* |
| TRIM62 | Low | 15 | 1 | 0.736 | ***0.001*** |  | 6 | 10 | -0.612 | ***0.003*** |
|  | High | 2 | 7 |  |  |  | 9 | 0 |  |  |

**Table S6** List of the antibodies used in this study

| Antibody name | Source |
| --- | --- |
| TRIM62 | Santa Cruz (sc-102144)/Abcam (ab51039) |
| β-actin | Sigma Aldrich (A5316) |
| α-catenin | Santa Cruz (sc-7894) |
| Vimentin | Santa Cruz (sc-6260) |
| c-Jun | Cell signaling (9165) |
| Slug | Cell signaling (9585) |
| JNK1 | Cell signaling (3708) |
| p-JNK1 | Cell signaling (4688) |
| CyclinD1 | Abcam (ab134175) |
| P27 | Abcam (ab32034) |

**Table S7** List of the reagents used in this study

| Reagent name | Source |
| --- | --- |
| Anti-Mouse IgG (whole molecule)–Peroxidase antibody produced in goat | Sigma (A5278) |
| Goat anti-Rabbit IgG-HRP Secondary Antibody | ZSGB-BIO (ZDR-5306) |
| Mitomycin C | Roche (M8170) |
| Phalloidin–Tetramethylrhodamine B isothiocyanate | Sigma (P1951) |
| GTVision^TM^III Polymer HRP Detection System | Gene Tech (GK500510A) |
| Hematoxylin and eosin (H&E) staining kit | Solarbio (G1120) |
| Cignal Finder Cancer 10-Pathway Reporter Array | QIAGEN (CCA-101L) |
